# MicroRNA transcriptome analysis reveals the potential role of miRNAs in regulating adipocyte hyperplasia and hypertrophy

**DOI:** 10.3389/fgene.2026.1737852

**Published:** 2026-01-16

**Authors:** Ligang Ni, Zhanpeng Gu, Xiaoyan Wang, Junsheng Zhang, Chunbao Zhou, Yaqin Zhang, Huizhen Gao, Pan Xu

**Affiliations:** 1 School of Animal Science and Technology, Jiangsu Agri-animal Husbandry Vocational College, Taizhou, China; 2 College of Animal Science and Technology, Yangzhou University, Yangzhou, China

**Keywords:** Sujiang pig, transcriptome profiling, backfat tissue, adipocyte hyperplasia, adipocyte hypertrophy

## Abstract

**Background:**

MicroRNAs (miRNAs) play a crucial role in regulating the development of backfat, yet their specific regulatory functions remain incompletely understood. The main objective of this study was to reveal the regulatory mechanism of miRNAs in porcine backfat development, and the molecular regulatory mechanisms of adipocyte hyperplasia and hypertrophy.

**Methods and results:**

In this study, we used high‐throughput sequencing technology to construct miRNA expression profiles of backfat tissue from Sujiang pigs at four developmental stages: 1, 3, 6, and 8 months old. A total of 292 known miRNAs and 274 novel miRNAs were identified. Through a comprehensive analysis of the miRNA and messenger RNA (mRNA) transcriptomes, we discovered many important mRNA‐miRNA pairs during porcine backfat development. Functional analysis revealed that the PI3K‐Akt signaling pathway, signaling pathways regulating pluripotency of stem cells, and mTOR signaling pathway as pivotal pathways related to adipocyte differentiation and hyperplasia. Butanoate metabolism, fatty acid metabolism, and fatty acid degradation were identified as pivotal pathways related to adipocyte metabolism and hypertrophy. Ssc‐miR‐1343, ssc‐miR‐1307, ssc‐miR‐1249, ssc‐miR‐331-3p, ssc‐miR‐296‐5p, ssc‐miR‐149, ssc‐miR‐361‐3p, and ssc‐miR‐615, which were identified as hub miRNAs in the miRNA‐mRNA regulatory network, may play crucial roles in regulating backfat development. Further validation revealed that ssc‐miR‐1343 can directly target the *TCF3* and *FASN* genes at different backfat developmental stages, potentially regulating adipocyte hyperplasia and hypertrophy.

**Conclusion:**

The comparative miRNA and mRNA transcriptomes analysis of porcine backfat tissue revealed the molecular regulatory mechanisms involved in porcine backfat development, providing useful information for the future breeding of pigs.

## 1 Introduction

The domestic pig (*Sus scrofa*) is a crucial species, contributing to over 37% of global meat consumption (McGlone, 2013). Carcass quality is an important economic trait in pig production, and a critical factor influencing carcass quality is the backfat thickness (Nevrkla et al., 2017). Backfat thickness is an essential indicator of fat deposition in pig production, and it also affects production efficiency and reproductive traits of pigs (China et al., 2019). Therefore, backfat thickness is a crucial breeding target in the pig industry, and studying the regulatory mechanism of backfat development could greatly benefit efficient pig production.

The development of backfat, which is a complex biological process regulated by multiple factors including nutrition, growing environment, and genetics, is characterized by adipocyte hyperplasia (increased numbers of adipocytes) and hypertrophy (enlarged adipocytes) (Etherton and Eugene, 1980). Genetics is widely recognized as a pivotal factor in porcine backfat development (Smith et al., 1991). MicroRNAs (miRNAs) are a class of endogenous non-coding single stranded RNAs with a length of 21–25 nucleotides. Functioning as key regulators of gene expression, these molecules can rapidly assemble into RNA-induced silencing complexes to post-transcriptionally modulate target genes. miRNAs play crucial roles in a wide range of fundamental biological processes in a living organism, including cell differentiation, metabolism, apoptosis, and immune responses (Hu et al., 2024; Shang et al., 2023). Previous research has shown that some miRNAs play crucial roles in regulating adipose tissue development. For instance, miR-143 promotes preadipocyte differentiation by directly targeting the *Pref-1* gene and inhibiting ERK1/2 pathway activation (Giammona et al., 2024; Kim et al., 2015). miR-455 specifically regulates brown adipogenesis through targeting *HIF1α*, which activates the AMPK–PGC1α pathway and subsequently promotes brown adipocyte differentiation as well as the expression of thermogenesis related genes (Zhang et al., 2015). Additionally, miR-23a exerts anti-adipogenic effects by downregulating adipocyte-specific genes and reducing lipid accumulation (Qi et al., 2016). miR-122 have been demonstrated to regulate lipid metabolism in adipocytes (Peixoto et al., 2021). While certain miRNAs have been shown to influence adipocyte differentiation and metabolism, few studies have investigated their roles in regulating adipocyte hyperplasia and hypertrophy during backfat development. Moreover, the molecular regulatory mechanisms underlying miRNA function in backfat development remain unclear.

With the rapid advancement of high-throughput sequencing technologies, particularly next-generation sequencing (NGS) and its continuous iteration, transcriptome sequencing has been extensively applied in animal genetic and breeding research (Banerjee et al., 2025). MicroRNA sequencing (miRNA-Seq) is an NGS-based technology that allows for highly sensitive, high-throughput profiling and quantification of miRNAs in biological samples, even at the single-cell level or in trace-volume exosomes (Benesova et al., 2021; Huang et al., 2023). It has greatly advanced our understanding of biological processes, physiological responses, and tissue development, especially in regulating animal economic traits such as fecundity, muscle growth, and fat deposition (Kappel and Keller, 2017). Additionally, miRNA-Seq has several prominent advantages, including enhanced sensitivity for detecting low-abundance miRNAs, the ability to identify novel and species-specific miRNAs, cost-effectiveness with the advancement of sequencing technology, and compatibility with multi-omics integration to construct comprehensive regulatory networks (Momin et al., 2021). These advantages have established miRNA-Seq as the predominant method in recent miRNA transcriptome studies aimed at animal genetic improvement. For example, integrated miRNA-mRNA profiling analysis of skeletal muscle from Jiangquan black pigs identified 330 negatively regulated miRNA-mRNA pairs, revealed miR-133 and miR-206 as critical regulators of muscle development, with miR-133 directly targeting the myogenic regulatory factor MSC (Gao et al., 2025). In a cattle genetic improvement study, miRNA-seq analysis of liver tissue from 60 steers (three breeds) identified significantly differentially expressed miRNAs (DE miRNAs), including bta-miR-449a and bta-miR-AB-2. These DE miRNAs target genes in lipid metabolism, insulin signaling, and mitochondrial function, offering insights for enhancing feed efficiency (Mukiibi et al., 2020).

The Sujiang pig is a synthetic breed that was created by crossing Chinese Jiangquhai pigs, Fengjing pigs, and Western Duroc pigs. This breed had undergone 12 generations of selective breeding since 2001. Compared with Chinese obese breeds and Western lean breeds, Sujiang pigs have moderate backfat thickness (Xu et al., 2020). They are considered to be good models for studying the molecular regulatory mechanisms of backfat development. In addition, studies have shown that porcine backfat development before 3 months of age is primarily driven by adipocyte hyperplasia. After 6 months of age, backfat development is predominantly characterized by adipocyte hypertrophy (Etherton and Eugene, 1980; Sun et al., 2000). In this study, we used miRNA-seq to construct the temporal miRNA expression profiles of backfat tissue samples collected from Sujiang pigs at four developmental stages (1, 3, 6, and 8 months old). DE miRNAs were identified across the four developmental stages and their target genes were analyzed. Furthermore, we examined mRNA-miRNA interactions to define a potential regulatory network involved in backfat development. The objective of this study was to gain a deeper understanding of the molecular regulatory mechanisms underlying adipocyte hyperplasia and hypertrophy during backfat development.

## 2 Materials and methods

### 2.1 Experimental pigs and tissue samples collection

Sujiang pigs (half-sib female) were used in this study, obtained from Sujiang pig breeding farm, Taizhou, China. All experimental pigs were raised under identical conditions, with *ad libitum* access to water and feed. Diets were formulated to meet nutritional requirements for pigs as suggested by the National Research Council (NRC, 2012). The ingredients and nutritional composition of the diets are shown in Supplementary Table S1. Three pigs each at 1, 3, 6, and 8 months of age (designated as M1, M3, M6, and M8) were selected based on similar body weight and live backfat thickness (measured at the last rib; see Supplementary Table S2). To minimize the suffering of experimental animals before slaughter, three pigs (1 month old) were anesthetized via intraperitoneal injection of xylazine (5 mg/kg), while the other selected pigs were euthanized by electrical stunning. After slaughter, backfat thickness was measured at the shoulder and 6th-7th rib to further determine the backfat development. Backfat tissue samples at the last rib were collected from the left carcass, with a portion immediately stored in liquid nitrogen and another portion fixed in 4% paraformaldehyde for later use.

### 2.2 Hematoxylin and eosin (H&E) staining

Backfat tissue was fixed in 4% paraformaldehyde at room temperature for 24 h. The fixed tissues were then washed three times with phosphate buffered saline (PBS) and cut into small pieces measuring approximately 1 cm × 0.5 cm × 1 cm. Paraffin sections were prepared from these pieces and stained with hematoxylin and eosin (H&E). The morphology of adipocytes was observed under an optical microscope.

### 2.3 RNA extraction and small RNA sequencing

Total RNA from twelve backfat tissue samples was extracted using TRIzol (Invitrogen, CA, United States) following the manufacturer’s protocol. RNA degradation and contamination were evaluated on 1.5% agarose gels. RNA concentration and integrity were measured using the Agilent 2100 Bioanalyzer (Agilent Technologies, CA, United States). RNA samples with an RNA Integrity Number (RIN) > 7.0 were selected for miRNA sequencing and were subsequently analyzed using the Illumina HiSeq 2500 platform.

### 2.4 Statistical analysis of the sequencing data

The raw data obtained by sequencing was checked and processed using fast quality control (FastQC) software (version 0.11.9). Clean reads were obtained by removing adaptor sequences, low quality contaminant reads, and discarding the sequences shorter than 17 nt or longer than 35 nt. Then, the clean reads were mapped to the reference genomic sequences of porcine (Sscrofa11.1), aligned to the Rfam database (http://rfam.xfam.org) and Repbase database (https://www.girinst.org/repbase), and used to identify non-coding RNA, including miRNA, ribosomal RNA (rRNA), transfer RNA (tRNA), small nuclear RNA (snRNA), small nucleolar RNA (snoRNA), etc. After excluding non-miRNA sequences, the remaining sequences were regarded as candidate miRNA reads. Known porcine miRNA sequences were searched in miRBase (http://www.mirbase.org) using Bowtie software (version 1.1.1), while any sequences that did not match any known miRNAs were further analyzed using miRdeep2 software (version 2.0.0.8) and predicted novel miRNAs.

### 2.5 Differential expression analysis and target gene prediction

The expression levels of known miRNAs and predicted novel miRNAs were normalized with transcripts per million (TPM) (Robinson and Oshlack, 2010). Principal Component Analysis (PCA) was performed to evaluate the effectiveness of batch effect correction and inter-dataset alignment. DE miRNAs were identified across stages (M3 vs. M1, M6 vs. M3, M8 vs. M6) using the edgeR package (http://www.r-project.org/). A false discovery rate (FDR) < 0.05 and a |log_2_(FoldChange)| > 1 were set as the threshold for determining significantly DE miRNAs. The target genes of these DE miRNAs were predicted using miRanda (http://www.microrna.org) and TargetScan (http://www.targetscan.org/). In a previous study, mRNA transcriptome profile was constructed (Ni et al., 2023). Predicted target genes of DE miRNAs were corresponded to the mRNA transcriptome profile, significant negatively correlated miRNA-mRNA pairs were evaluated using the Pearson correlation coefficient (PCC) (PCC < −0.7 and *p*-value < 0.05).

### 2.6 Validation of differentially expressed miRNAs

To verify the miRNA sequencing data, we conducted stem-loop reverse transcription quantitative polymerase chain reaction (RT-qPCR) to confirm the expression levels of specific miRNAs in porcine backfat tissues. Four DE miRNAs were randomly selected for validation, using porcine U6 snRNA as an endogenous control. The primers used for RT-qPCR are listed in Supplementary Table S3. The RNA samples used for RT-qPCR were the same as those used for miRNA sequencing. RT-qPCR was performed on a LightCycler 480 real-time PCR system (Roche Diagnostics, Mannheim, Germany), all reactions were performed in triplicate, and the miRNA expression levels were calculated using the 2^−ΔΔCT^ method.

### 2.7 Functional enrichment analysis and integrated miRNA-mRNA network analysis

To annotate the biological functions of DE miRNAs, the predicted target genes in the miRNA-mRNA pairs were subjected to Kyoto Encyclopaedia of Genes and Genomes (KEGG) pathway enrichment analysis. The results were visualized using the clusterProfiler *R* package, and with a *p*-value < 0.05 considered as significantly enriched KEGG pathways. Subsequently, miRNA-mRNA interaction networks were constructed using Cytoscape software (http://www.cytoscape.org/) to accurately identify the key associations between DE miRNAs and significantly differentially expressed genes (DE genes).

### 2.8 miRNA-target identification and dual-luciferase reporter assay

The targeting binding sites of ssc-miR-1343 in *TCF3* and *FASN* were predicted using RNAhybrid (version 2.2). The fragments of the 3′untranslated region (UTR) of *TCF3* and *FASN*, which contain the putative wild-type (Wt) or mutated (Mut) ssc-miR-1343 binding sites, were chemically synthesized and inserted into pmirGLO vector to obtain two wild-type vectors (pmirGLO-*TCF3*-3′UTR-Wt, pmirGLO-*FASN*-3′UTR-Wt) and two mutated vectors (pmirGLO-*TCF3*-3′UTR-Mut, pmirGLO-*FASN*-3′UTR-Mut). HEK293T cells were cultured and co-transfected with wild-type vectors or mutated vectors, along with ssc-miR-1343 mimic or negative control (NC) mimic using Lipofectamine 2000 reagent for 48 h. Then, luciferase activities were measured using the dual-luciferase reporter assay system. All nucleic acid sequences were synthesized by Shanghai GenePharma Co., Ltd., Shanghai, China.

## 3 Results

### 3.1 Developmental changes of backfat across different stages

**FIGURE 1:**
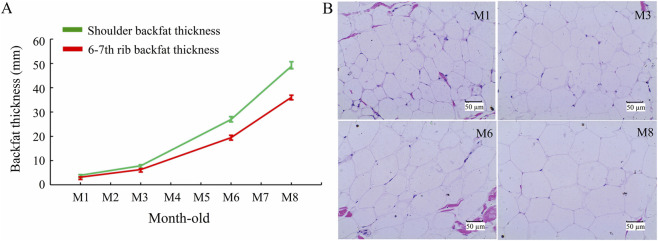
Developmental changes in porcine backfat. **(A)** The trend graph of backfat thickness change during backfat development; **(B)** H&E staining of backfat adipocytes in the four developmental stages, scale bars indicate 50 µm.

In this research, the shoulder backfat thickness and 6th-7th rib backfat thickness of Sujiang pigs were measured at different developmental stages (M1, M3, M6, M8). The results showed a gradual increase in both types of backfat thickness over time, with a slow increase before 3 months old, and a rapid increase from the M6 to M8 stage (Figure 1A). H&E staining also showed that the diameter of backfat adipocytes slowly enlarged before 3 months of age, with a significant enlarge from stage M6 to M8 (Figure 1B). This suggests that backfat development can be characterized by time.

### 3.2 Analysis of small RNA sequencing libraries

**FIGURE 2:**
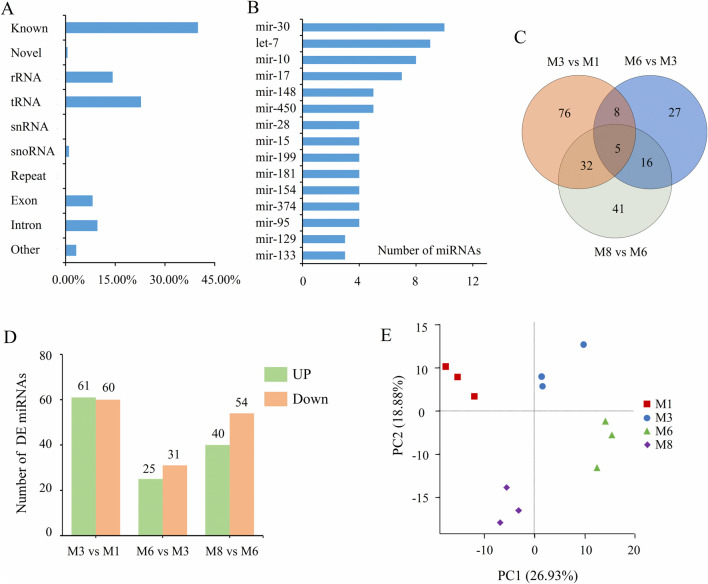
Characterization of miRNA expression profiles during backfat development. **(A)** Rfam classification of the mapped clean reads; **(B)** miRNA families of the identified miRNAs; **(C)** Venn diagrams show the number of DE miRNAs across different stages; **(D)** Number of upregulated and downregulated DE miRNAs across different stages; **(E)** PCA score plot for all samples of four stages.

To investigate the expression of small RNAs during the development of backfat in Sujiang pigs, we constructed twelve small RNA libraries from four different stages (M1, M3, M6, M8), with each stage having three small RNA libraries. The small RNA sequencing results are summarized in Supplementary Table S4, a total of 11,480,184–23,999,586 raw reads were generated from the twelve libraries, of which 8,214,171–18,219,981 clean reads were obtained. Furthermore, 95.32%–97.15% of the clean reads could be mapped to the reference genome. Among the mapped clean reads, an average of 14.24% were identified as rRNAs, 22.79% as tRNAs, 0.17% as snRNAs, and 1.05% as snoRNAs (Figure 2A).

### 3.3 Differential expression analysis of miRNAs

In the twelve libraries, known miRNAs were identified by searching in miRbase and a total of 292 miRNAs were detected (Supplementary Table S5). 274 novel miRNAs were predicted using miRDeep2 (Supplementary Table S6). Two known miRNAs (ssc-miR-99a, ssc-miR-10b) had over 5,000,000 reads each, and 13 known miRNAs (ssc-miR-26a, ssc-miR-199b-3p, ssc-miR-148a-3p, ssc-miR-199a-5p, ssc-miR-143-3p, etc.) had over 1,000,000 reads each. The most abundant miRNA families were miR-30 (10 members), let-7 (9 members), miR-10 (8 members), and miR-17 (7 members) (Figure 2B). PCA of the normalized miRNA expression levels revealed that samples were grouped according to the four stages (Figure 2E).

Subsequently, three comparisons (M3 vs. M1, M6 vs. M3, M8 vs. M6) were investigated, a total of 205 DE miRNAs were identified. Among these DE miRNAs, 121 DE miRNAs were identified in the M3 vs. M1 comparison, with 60 upregulated and 61 downregulated. 56 DE miRNAs were identified in M6 vs. M3, with 25 upregulated and 31 downregulated. 94 DE miRNAs were identified in M8 vs. M6, with 40 upregulated and 54 downregulated (Figure 2D). A Venn diagram analysis showed that the three pairwise comparisons shared 5 common DE miRNAs (Figure 2C), including ssc-miR-149, ssc-miR-338, ssc-miR-124a, ssc-novel-146, and ssc-novel-109.

### 3.4 Validation of miRNA expression by RT-qPCR

**FIGURE 3:**
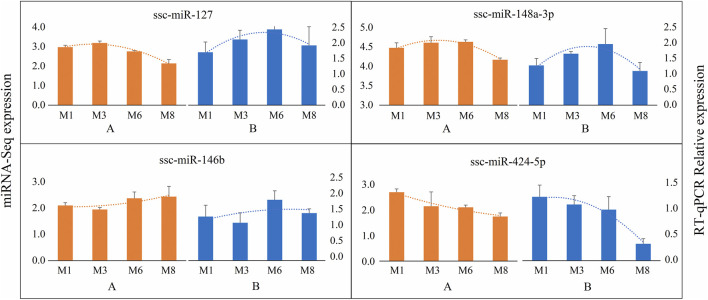
Expressions of the DE miRNAs using miRNA-Seq and RT-qPCR. **(A)** DE miRNAs expression as detected by miRNA-Seq; **(B)** RT-qPCR analysis of selected DE miRNAs. Error bars indicate the mean ± standard deviation of three independent replicates.

Four DE miRNAs (ssc-miR-127, ssc-miR-146b, ssc-miR-148a-3p, ssc-miR-424-5p) were selected for validation through RT-qPCR. The expression patterns observed were consistent with the miRNA-Seq results (Figure 3). These results confirm the reliability of the miRNA-Seq data and its suitability for subsequent analysis.

### 3.5 Target gene prediction of DE miRNAs

Target genes were predicted using TargetScan and miRanda. This analysis identified 8,117 potential target genes for the 121 DE miRNAs in the M3 vs. M1 comparison, 2,789 for the 56 DE miRNAs in M6 vs. M3, and 6,836 for the 94 DE miRNAs in M8 vs. M6. An integrated analysis of the mRNA and miRNA expression profiles revealed 2435 mRNA-miRNA pairs, 91 mRNA-miRNA pairs, and 1215 mRNA-miRNA pairs in the three comparisons (Supplementary Table S7), respectively.

### 3.6 Enrichment analysis of DE miRNAs

**FIGURE 4:**
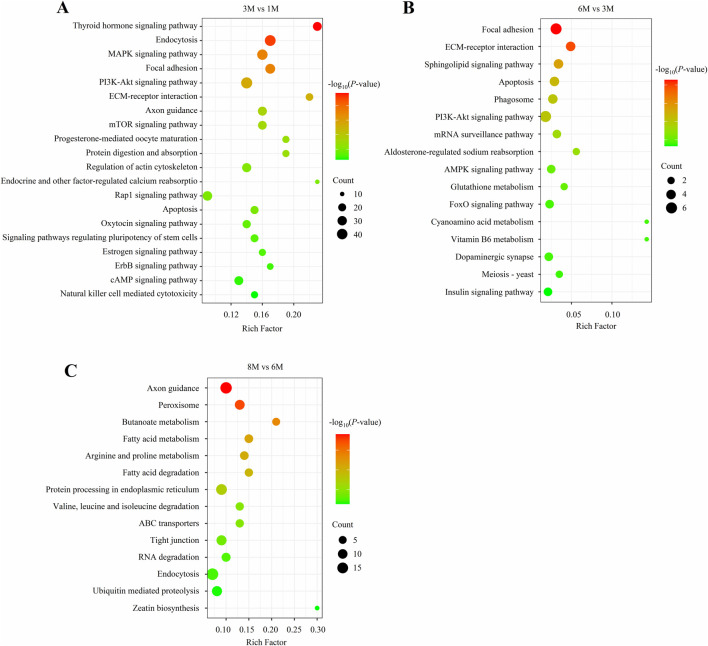
The top 20 significant KEGG pathways of miRNA-targeted mRNAs in the three comparisons. **(A)** KEGG pathways in the M3 vs. M1 comparison; **(B)** KEGG pathways in the M6 vs. M3 comparison; **(C)** KEGG pathways in the M8 vs. M6 comparison.

Functional enrichment analysis was performed on the miRNA-targeted mRNAs in the three comparisons. The top 20 significant KEGG pathways for the three combinations are shown in Figure 4. The result revealed that many pathways associated with adipocyte differentiation and hyperplasia, such as the PI3K-Akt signaling pathway, pathways regulating pluripotency of stem cells, and the mTOR signaling pathway, were significantly activated in the M3 vs. M1 comparison (Figure 4A). In contrast, many pathways related to adipocyte metabolism and hypertrophy were significantly activated in the M8 vs. M6 comparison, these included butanoate metabolism, fatty acid metabolism, and fatty acid degradation (Figure 4B). However, for the M6 vs. M3 comparison, apart from the pathways related to adipocyte differentiation and hyperplasia (e.g., the PI3K and FoxO signaling pathways) were significantly activated, few pathways related to adipocyte metabolism and hypertrophy (e.g., the sphingolipid and insulin signaling pathways) were also significantly activated (Figure 4C). These results suggest that miRNA-targeted mRNAs play crucial roles in regulating adipocyte hyperplasia and hypertrophy during backfat development.

### 3.7 miRNA-mRNA networks involved during backfat development

**FIGURE 5:**
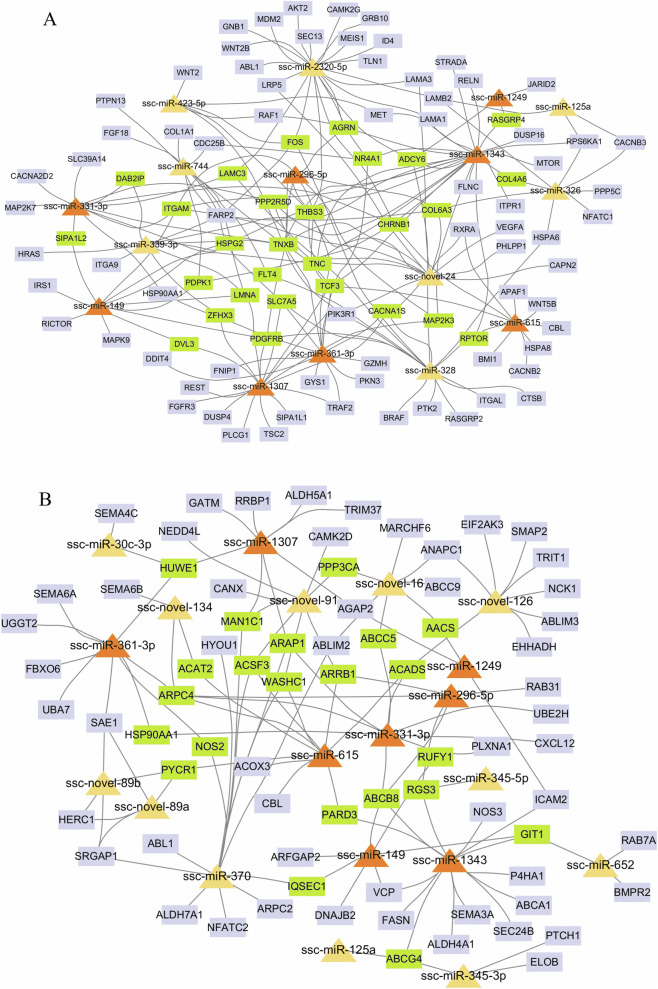
Visualization of miRNA-mRNA regulatory networks. **(A)** miRNA-mRNA interaction network associated with adipocyte differentiation and hyperplasia; **(B)** miRNA-mRNA interaction network associated with adipocyte metabolism and hypertrophy. Shared hub miRNAs in the two networks are highlighted in orange, while critical node genes are marked in green.

To further narrow down the list of miRNAs involved in porcine backfat development, we focused on key miRNA-mRNA pairs. Based on the results of the enrichment analysis, 185 miRNA-mRNA pairs closely related to adipocyte differentiation and hyperplasia were screened, and 122 miRNA-mRNA pairs closely related to adipocyte metabolism and hypertrophy were screened (Supplementary Table S8). Subsequently, miRNA-mRNA regulatory networks were constructed using Cytoscape software. The results showed that the two miRNA-mRNA networks shared several hub miRNAs, including ssc-miR-1343, ssc-miR-1307, ssc-miR-1249, ssc-miR-331-3p, ssc-miR-296-5p, ssc-miR-149, ssc-miR-361-3p and ssc-miR-615. Critical node genes related to adipocyte differentiation and hyperplasia were found in the network A (Figure 5A), including *ADCY6*, *CHRNB1*, *SLC7A5*, *TCF3*, *MAP2K3*, *COL6A3*, *ITGAM*, *CACNA1S*, *THBS3*, *TNXB*, etc. Critical node genes related to related to adipocyte metabolism and hypertrophy were found in the network B (Figure 5B), including *AACS*, *ABCB8*, *ACADS*, *ACSF3*, *ABCG4*, *ARPC4*, *MAN1C1*, *WASHC1*, etc.

### 3.8 Important miRNA-mRNA pairs and pathways

**FIGURE 6:**
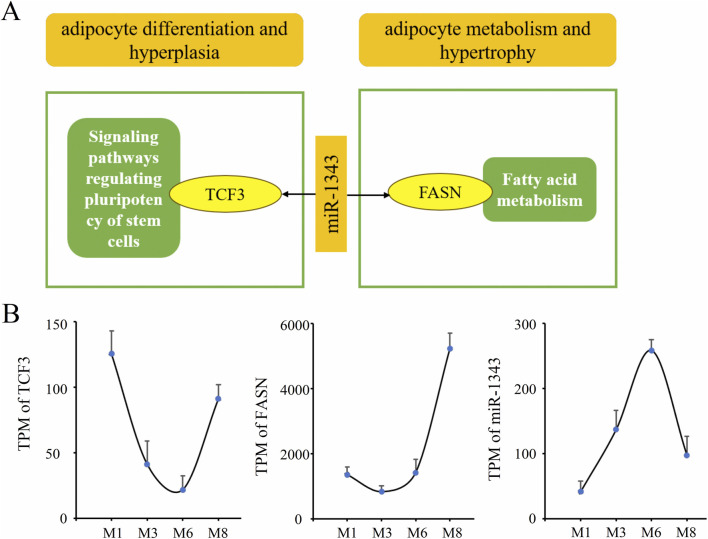
Important miRNA-mRNA pairs involved in adipocyte hyperplasia and hypertrophy. **(A)** Important miRNA-mRNA pairs and regulatory pathways; **(B)** Expression pattern of *TCF3*, *FASN*, and ssc-miR-1343 during backfat development.

**FIGURE 7:**
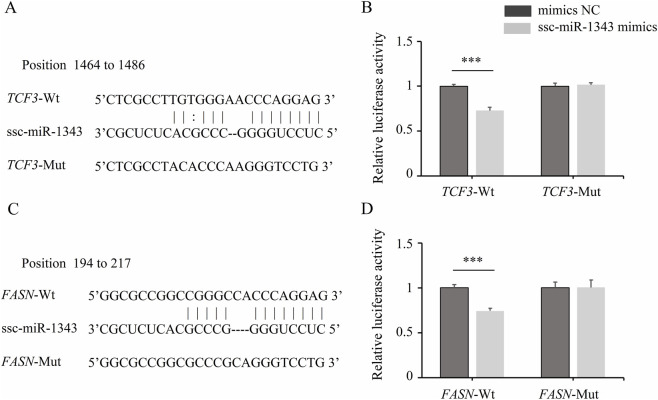
Validation of *TCF3* and *FASN* as direct targets of ssc-miR-1343. **(A)** The information of ssc-miR-1343 and *TCF3* interaction and their binding sites; **(B)** Validation of the targeting relationship between ssc-miR-1343 and *TCF3* by using dual-luciferase reporter assay; **(C)** The information of ssc-miR-1343 and *FASN* interaction and their binding sites; **(D)** Validation of the targeting relationship between ssc-miR-1343 and *FASN* by using dual-luciferase reporter assay. Error bars indicate the mean ± standard deviation of triplicate experiments, *** indicate *p*-value < 0.001.

In two miRNA-mRNA regulatory networks, ssc-miR-1343 targets the most abundant mRNAs, with 25 mRNAs in network A and 14 mRNAs in network B. Two important pairs (miR-1343-*TCF3* and miR-1343-*FASN*) were found to be involved in pathways related to adipocyte hyperplasia and hypertrophy (Figure 6A). The binding sites of ssc-miR-1343 to the 3′UTR of *TCF3* and *FASN* was predicted and shown in Figures 7A,C. The results of dual-luciferase reporter assay (Figures 7B,D) showed that ssc-miR-1343 can significantly decrease the luciferase activity from the wild-type vector plasmids (pmirGLO-*TCF3*-3′UTR-Wt, pmirGLO-*FASN*-3′UTR-Wt) compared to the NC control (*p*-value < 0.05) and failed to decrease the luciferase activity from the mutated vectors plasmid (pmirGLO-*TCF3*-3′UTR-Mut, pmirGLO-*FASN*-3′UTR-Mut). Furthermore, expression pattern analysis showed that the expression of ssc-miR-1343 did not show a positive correlation with the growth trajectory of backfat thickness. It exhibited increased expression from the M1 to M3 stage, plateaued between M3 and M6 stage, and then declined from the M6 to M8 stage. The expression patterns of *TCF3* and *FASN* were essentially negatively correlated with that of ssc-miR-1343, with the exception of *FASN* during the M3 to M6 stage (Figure 6B).

## 4 Discussion

Backfat thickness is a crucial trait in pigs that greatly affects the quality of pork carcass and meat quality (Nevrkla et al., 2017). The backfat thickness is mainly determined by the development of adipocytes in backfat tissue, which undergo both hyperplasia and hypertrophy (Etherton and Eugene, 1980). In pigs, the hyperplastic development of backfat adipocytes mainly occurs before 3 months of age and continues until at least 6 months. As pigs grow, the intensity of adipocyte hypertrophy increases, with the most significant hypertrophy occurring after the age of 6 months (Etherton and Eugene, 1980; Campbell and Dunkin, 1983). Recent studies have revealed that specific genes, including *IGF2* and *KLF7*, are significantly upregulated in the adipose tissue of pigs before 3 months of age, thereby activating the PI3K-Akt signaling pathway and promoting the proliferation and differentiation of adipocytes. However, after 6 months of age, genes such as *LPL*, *PPARγ*, and *C/EBPα* exhibit sustained upregulation in the adipose tissue, thereby enhancing fat deposition and driving adipocyte hypertrophy (Liu et al., 2018; Liu et al., 2024; Pan et al., 2017). Consistently, studies have demonstrated that adipocyte hypertrophy is the main factor influencing backfat thickness, whereas the impact of adipocyte hyperplasia is relatively limited (Nakajima et al., 2011). This study on Sujiang pigs showed that the diameter of adipocytes in backfat tissue enlarged slowly before 3 months old, but increased rapidly from the M6 to M8 stage. Similarly, the backfat thickness also increased slowly before 3 months old, and rapidly from the M6 to M8 stage. These results clearly demonstrate that in porcine backfat tissue, adipocytes undergo hyperplasia primarily before 3 months old, whereas hypertrophy becomes the dominant process after 6 months.

miRNAs are considered among the most important regulatory factors in animals. Previous research has found that they are involved in almost all biological processes, such as cell differentiation, hypertrophy, metabolism, and hyperplasia (Hu et al., 2024; Shang et al., 2023). To analyze the molecular regulatory mechanisms involved in backfat development, we used high-throughput sequencing technology to construct miRNA expression profiles of backfat tissue from Sujiang pigs at four different developmental stages: 1, 3, 6, and 8 months old. In this study, a total of 292 known miRNAs and 274 novel miRNAs were identified. Most mature miRNAs belonged to the miR-30, let-7, miR-10, and miR-17 families, which is consistent with previous studies (Li et al., 2011). The most abundant miRNAs were ssc-miR-99a, ssc-miR-10b, ssc-miR-26a, ssc-miR-199b-3p, ssc-miR-148a-3p, ssc-miR-199a-5p, and ssc-miR-143-3p. These miRNAs have been reported to have regulatory functions during adipose tissue development. For example, studies have shown that ssc-miR-99a is expressed at low levels in young pig subcutaneous adipose tissue and is involved in adipogenesis during preadipocyte differentiation (Khatoon et al., 2014). Ssc-miR-199a-5p has been found to either attenuate cell proliferation or promote lipid deposition in porcine preadipocytes (Shi et al., 2014). Overexpression of ssc-miR-143-3p in yak adipose tissue has been shown to either inhibit or promote adipocyte differentiation and hypertrophy at different developmental stages (Ji et al., 2020). Collectively, these findings suggest that the miRNAs identified in this study have important regulatory functions during backfat development.

Differential expression analysis of miRNAs across stages revealed that the DE miRNAs exhibited developmental stage specificity. A total of 121 DE miRNAs were identified in the M3 vs. M1 comparison, 94 DE miRNAs identified in M8 vs. M6 comparison, and only 56 DE miRNAs identified in M6 vs. M3 comparison. The three pairwise comparisons shared 5 common DE miRNAs, including three known miRNAs, ssc-miR-149, ssc-miR-338, and ssc-miR-124a. Ssc-miR-149 was upregulated in the M3 vs. M1 comparison but downregulated in the other two pairwise comparisons. Ssc-miR-149 has been identified as a key regulator in various signaling pathways, such as AMPK, Wnt, and Jak/STAT. In porcine skeletal muscle, it plays an important role in regulating cell proliferation and differentiation by targeting the *PIK3CD* gene, while it may also be implicated in the regulation of intramuscular fat (IMF) deposition (Qi et al., 2022). Ssc-miR-338 was upregulated in the three pairwise comparisons and has been implicated in fat metabolism. In porcine backfat tissue, it regulates lipid deposition by targeting the *FASN* gene (Xing et al., 2019). The results indicate that miRNAs with distinct functions may play important roles in specific stages of porcine backfat development.

To investigate the function of DE miRNAs, an integrated analysis of the mRNA and miRNA expression profiles was performed by combining previous mRNA transcriptome data (Ni et al., 2023). Functional enrichment analysis was performed on the targeted genes in the miRNA-mRNA pairs. The results revealed that the PI3K-Akt signaling pathway, signaling pathways regulating pluripotency of stem cells, and mTOR signaling pathway were significantly activated in M3 vs. M1 comparison. Previous studies have shown that these pathways play an important role in regulating adipocyte differentiation and hyperplasia (Gao et al., 2018; Chen et al., 2013; Beretta et al., 2015). In contrast, Butanoate metabolism, Fatty acid metabolism, and Fatty acid degradation were significantly activated in M8 vs. M6 comparison. These pathways have been proven to be important in regulating adipocyte metabolism and hypertrophy (Wolfrum and Spener, 2000; Swiątkiewicz et al., 2016). However, for the M6 vs. M3 comparison, only a few pathways related to adipocyte metabolism and hypertrophy were activated, such as the Sphingolipid signaling pathway (Ding et al., 2019) and Insulin signaling pathways (You et al., 2017). Additionally, some pathways related to adipocyte differentiation and hyperplasia were also activated, such as the PI3K-Akt signaling pathway (Swiątkiewicz et al., 2016) and FoxO signaling pathway (Jiang et al., 2019). These results suggest that adipocyte differentiation and hyperplasia mainly occur during the early developmental stage (before 3 months of age) of porcine backfat, while adipocyte metabolism and hypertrophy mainly take place during the late developmental stage (after 6 months of age).

To gain a deeper understanding of the roles of DE miRNAs in backfat development, we constructed two miRNA-mRNA regulatory networks. Network A is associated with adipocyte differentiation and hyperplasia, while Network B is associated with adipocyte metabolism and hypertrophy. Certain hub genes have been shown to play significant roles in regulating adipocyte proliferation and hyperplasia during the early stages of backfat development (Network A). For instance, *SLC7A5*, a member of the solute carrier (SLC) family, is responsible for transporting amino acids into cells and controlling cell proliferation and development (Sokolov et al., 2020). Additionally, the expression of *COL6A3* is regulated by *ATF3* in preadipocytes and is involved in cell differentiation and proliferation (Saraf et al., 2013). Similarly, specific hub genes have been found to have important functions in regulating adipocyte metabolism and hypertrophy during the later stages of backfat development (Network B). For example, *ACADS* is a flavoenzyme gene that catalyzes the α,β-dehydrogenation of acyl-CoA esters (Swigonova et al., 2009) and plays a crucial role in free fatty acid beta-oxidation and metabolism, as well as in regulating energy homeostasis (Chen and Su, 2015). Another enzyme gene, *ACSF3*, functions by catalyzing the formation of thioesters between fatty acids and coenzyme A, and helps regulate intracellular free fatty acid levels as well as contributing to the regulation of lipid metabolism (Ratnavadivel et al., 2022). Additionally, the expression of *ABCG4* has been shown to affect insulin responsiveness in pancreatic beta cells and promote lipid metabolism and adipocyte hypertrophy (Huang et al., 2021).

Interestingly, these two networks share several DE miRNAs, including ssc-miR-1343, ssc-miR-1307, ssc-miR-331-3p, ssc-miR-361-3p, and ssc-miR-615. However, these DE miRNAs target different genes involved in the regulation of backfat development. For example, ssc-miR-1249 potentially targets *ADCY6*, *RASGRP4*, and *JARID2* in Network A, while it potentially targets *AACS*, *AGAP2*, and *ICAM2* in Network B. *JARID2* belongs to the Jumonji family of proteins, whose members are involved in the regulation of embryonic stem cell differentiation and proliferation (Mejetta et al., 2011). *RasGRP4* is highly expressed in mast cells and plays an important role in their differentiation and proliferation (Adachi et al., 2012). Additionally, *AACS* is a ketone body-utilizing enzyme gene that, when overexpressed in subcutaneous adipocytes, can promote fatty acid synthesis (Yamasaki et al., 2007). The expression of *AGAP2* in liver tissue can affect the expression of several genes involved in lipid, bile acid, and fatty acid metabolism (Pedersen et al., 2019).

Similarly, ssc-miR-1343 potentially targets different genes in Network A and Network B. Previous studies have shown that ssc-miR-1343 is expressed in porcine backfat tissue and exhibits differential expression between the fat-type Meishan and lean-type Large White pigs, implicating it as a potential regulator of backfat thickness (Chen et al., 2012). In this study, miRNA-mRNA network analysis revealed that ssc-miR-1343 targets two key adipogenic genes (*TCF3* and *FASN*) at different developmental stages of backfat. Dual-luciferase reporter gene assays demonstrated that *TCF3* and *FASN* directly bind with ssc-miR-1343. *TCF3*, a member of the Tcf/Lef family, functions as a transcriptional repressor (Nam et al., 2015). Furthermore, the ablation of *TCF3* function can enhance cell differentiation and proliferation (Atlasi et al., 2013). In this study, *TCF3* was downregulated in the M3 vs. M1 comparison and had a significant negative relationship with ssc-miR-1343, suggesting that ssc-miR-1343 may promote adipocyte differentiation and hyperplasia by inhibiting *TCF3* expression during the early developmental stage. Additionally, *FASN* is a lipogenic enzyme that plays an important role in regulating fatty acid synthesis in pigs (Liu et al., 2024). Studies have shown that upregulation of *FASN* expression drives adipocyte hypertrophy, identifying it as a candidate gene associated with backfat thickness in Landrace and Large White pigs (Chen et al., 2015; Grzes et al., 2016). In this study, *FASN* was upregulated in the M8 vs. M6 comparison and had a significant negative relationship with ssc-miR-1343, suggesting that ssc-miR-1343 may promote fatty acid synthesis and adipocyte hypertrophy by regulating *FASN* expression during the late developmental stage. Based on this, we propose that ssc-miR-1343 may regulate adipocyte hyperplasia and hypertrophy by targeting *TCF3* and *FASN*. These findings suggest that ssc-miR-1343 and its target genes represent a previously unknown mechanism regulating fat deposition in porcine backfat, with potential applications in breeding for reduced backfat thickness. However, this study only identified potential regulatory molecules in backfat tissue at different stages of development. Further genetic experiments are required to confirm their functional roles in fat deposition and determine their regulatory effects on backfat thickness.

In conclusion, this study conducted an integrated analysis of miRNA and mRNA transcriptomes of backfat tissue in order to uncover the molecular regulatory mechanisms of porcine backfat development. Through this analysis, we identified several candidate miRNAs that are associated with backfat development. Among these candidates, ssc-miR-1343 emerged as a crucial regulator, targeting *TCF3* and *FASN* at different stages and potentially regulating adipocyte hyperplasia and hypertrophy. This study presents a novel approach for using miRNAs and their target genes to regulate fat deposition in porcine backfat tissue.

## Data Availability

The datasets for this study have been deposited in the NCBI Sequence Read Archive under accession number PRJNA1107832 (https://www.ncbi.nlm.nih.gov/bioproject/PRJNA1107832/).
