# Supplementary material for: MicroRNA transcriptome analysis reveals the potential role of miRNAs in regulating adipocyte hyperplasia and hypertrophy
Source: Front Genet. 2026 Jan 16;17:1737852. doi: 10.3389/fgene.2026.1737852 (PMC12856495; doi:10.3389/fgene.2026.1737852)
Supplement: Supplementary file 1 [file Table1.docx]

**Supplementary Table S1** The ingredients and nutritional composition of the diets (air-dry basis) %

| **Items** | **Growing stage** | | **Finishing stage** | |
| --- | --- | --- | --- | --- |
|  | **8 to 30 kg** | **30 to 60 kg** | **60 kg to 90kg** | **90 kg to 120 kg** |
| Ingredients |  |  |  |  |
| Corn | 60.00 | 62.00 | 64.50 | 65.00 |
| Soybean meal | 19.30 | 18.60 | 15.80 | 13.50 |
| Fish meal | 4.50 | 0.00 | 0.00 | 0.00 |
| Wheat bran | 7.00 | 9.00 | 9.60 | 10.00 |
| Rice bran | 3.50 | 5.00 | 5.00 | 6.65 |
| Soybean oil | 2.00 | 2.00 | 2.00 | 2.00 |
| *L*-lysine-HCl | 0.50 | 0.25 | 0.20 | 0.15 |
| *DL-*methionine | 0.20 | 0.15 | 0.10 | 0.07 |
| *L*-threonine | 0.20 | 0.15 | 0.05 | 0.03 |
| *L*-tryptophan | 0.05 | 0.00 | 0.00 | 0.00 |
| Limestone (CaCO3) | 0.80 | 1.05 | 1.00 | 0.85 |
| Dicalcium phosphate | 0.65 | 0.50 | 0.45 | 0.45 |
| Salt | 0.30 | 0.30 | 0.30 | 0.30 |
| Premix^1^ | 1.00 | 1.00 | 1.00 | 1.00 |
| Total | 1. 0 | 100.00 | 100.00 | 100.00 |
| Nutrient levels^2^ |  |  |  |  |
| Digestible energy, MJ/kg | 13.73 | 13.71 | 13.72 | 13.71 |
| Crude protein | 17.70 | 15.23 | 14.64 | 13.90 |
| Crude fiber | 2.12 | 2.70 | 3.10 | 3.32 |
| Total Ca | 0.62 | 0.59 | 0.54 | 0.50 |
| Total P | 0.55 | 0.52 | 0.49 | 0.48 |

1 The premix provided the following per kilogram of the diet: vitamin A, 6,000 IU; vitamin D_3_, 400 IU; vitamin E, 10 IU; vitamin K_3_, 2 mg; vitamin B_1_, 1 mg; vitamin B_2_, 6.4 mg; vitamin B_6_, 2.4 mg; vitamin B_12_, 0.02 mg; vitamin C, 20 mg; biotin, 0.2 mg; folic acid, 0.2 mg; nicotinic acid, 14 mg; *D*-pantothenic acid, 10 mg; Cu (as copper sulfate), 6 mg; Fe (as ferrous sulfate), 60 mg; Mn (as manganese sulfate), 10 mg; Zn (as zinc sulfate), 60 mg; I (as potassium iodide), 0.3 mg; and Se (as sodium selenite), 0.3 mg.

2 The calculated values for digestible energy and analyzed values for other nutrients.
