# Supplementary material for: MicroRNA transcriptome analysis reveals the potential role of miRNAs in regulating adipocyte hyperplasia and hypertrophy
Source: Front Genet. 2026 Jan 16;17:1737852. doi: 10.3389/fgene.2026.1737852 (PMC12856495; doi:10.3389/fgene.2026.1737852)
Supplement: Supplementary file 3 [file Table2.docx]

**Supplementary Table S2** Weight and live backfat thickness of experimental pigs

| **Groups** | **Experimental pigs** | **Weight/kg** | **Live backfat thickness/mm** |
| --- | --- | --- | --- |
| M1 | M1-1 | 7.52 | --- |
|  | M1-2 | 7.28 | --- |
|  | M1-3 | 7.43 | --- |
| M3 | M3-1 | 34.04 | 7.20 |
|  | M3-2 | 35.25 | 7.10 |
|  | M3-3 | 34.85 | 7.50 |
| M6 | M6-1 | 79.50 | 15.90 |
|  | M6-2 | 83.40 | 16.20 |
|  | M6-3 | 81.10 | 16.50 |
| M8 | M8-1 | 114.40 | 23.50 |
|  | M8-2 | 111.50 | 22.90 |
|  | M8-3 | 115.80 | 23.40 |

Live backfat thickness were measured at last rib; At M1 stage, the live backfat thickness of experiment pigs was not tested due to their thin backfat.
