# Supplementary material for: MicroRNA transcriptome analysis reveals the potential role of miRNAs in regulating adipocyte hyperplasia and hypertrophy
Source: Front Genet. 2026 Jan 16;17:1737852. doi: 10.3389/fgene.2026.1737852 (PMC12856495; doi:10.3389/fgene.2026.1737852)
Supplement: Supplementary file 4 [file Table3.docx]

**Supplementary Table S3** Primer sequences used in RT-qPCR

| **Name of miRNA** | **Primer** | **Sequences (5'-3’)** |
| --- | --- | --- |
| ssc-miR-127 | RT | CTCAACTGGTGTCGTGGAGTCGGCAATTCAGTTGAG AGCCAAGC |
|  | FW | ACACTCCAGCTGGGTCGGATCCGTCTGA |
| ssc-miR-146b | RT | CTCAACTGGTGTCGTGGAGTCGGCAATTCAGTTGAGGCCTATGG |
|  | FW | ACACTCCAGCTGGGTGAGAACTGAATTC |
| ssc-miR-148a-3p | RT | CTCAACTGGTGTCGTGGAGTCGGCAATTCAGTTGAGACAAAGTTCT |
|  | FW | ACACTCCAGCTGGGTCAGTGCACTACAG |
| ssc-miR-424-5p | RT | CTCAACTGGTGTCGTGGAGTCGGCAATTCAGTTGAGTTCAAAACAT |
|  | FW | ACACTCCAGCTGGGCAGCAGCAATTCAT |
|  | URP* | TGGTGTCGTGGAGTCG |
| U6 snRNA | RT# | CAAACACCCAGTGACTTCCTAAA |
|  | FW | CCGTCTGTTCCAAGGCTATG |

RT: Reverse transcription primer; FW: Forward primer.

^*^ The universal reverse primer.

^#^ Also as the reverse primer of U6 snRNA.
