# Supplementary material for: MicroRNA transcriptome analysis reveals the potential role of miRNAs in regulating adipocyte hyperplasia and hypertrophy
Source: Front Genet. 2026 Jan 16;17:1737852. doi: 10.3389/fgene.2026.1737852 (PMC12856495; doi:10.3389/fgene.2026.1737852)
Supplement: Supplementary file 5 [file Table4.docx]

**Supplementary Table S4** The small RNA sequencing data and mapping to the reference genome

| **Sample** | **Raw reads** | **Clean reads** | **Total reads mapped** | **Unique mapped** |
| --- | --- | --- | --- | --- |
| M1-1 | 23999586 | 18219981 | 17583355(96.51%) | 3103737(17.65%) |
| M1-2 | 20791305 | 16265670 | 15715167(96.62%) | 2480270(15.78%) |
| M1-3 | 14296481 | 11958306 | 11541243(96.51%) | 1926615(16.69%) |
| M3-1 | 12310784 | 9770861 | 9427970(96.49%) | 1280311(13.58%) |
| M3-2 | 12743796 | 9427336 | 9063538(96.14%) | 767659(8.47%) |
| M3-3 | 11480184 | 8214171 | 7980392(97.15%) | 423872(5.31%) |
| M6-1 | 17895351 | 14151248 | 13551649(95.76%) | 1856448(13.70%) |
| M6-2 | 12791136 | 11068201 | 10653046(96.25%) | 425967(4.00%) |
| M6-3 | 13433835 | 9305919 | 8958909(96.27%) | 1770626(19.76%) |
| M8-1 | 15142900 | 13042331 | 12578235(96.44%) | 1469367(11.68%) |
| M8-2 | 11924901 | 10043980 | 9574378(95.32%) | 1564896(16.34%) |
| M8-3 | 16537544 | 14162164 | 13630864(96.25%) | 2015248(14.78%) |
